# Preclinical models of pancreatic ductal adenocarcinoma: challenges and opportunities in the era of precision medicine

**DOI:** 10.1186/s13046-020-01787-5

**Published:** 2021-01-05

**Authors:** Yiqi Yu, Gang Yang, Hua Huang, Ziyao Fu, Zhe Cao, Lianfang Zheng, Lei You, Taiping Zhang

**Affiliations:** 1 Department of General Surgery, Peking Union Medical College Hospital, Chinese Academy of Medical Sciences and Peking Union Medical College, Beijing, 100730 China; 2 Chinese Academy of Medical Sciences and Peking Union Medical College, Beijing, 100730 China; 3 Department of Nuclear Medicine, Peking Union Medical College Hospital, Chinese Academy of Medical Sciences and Peking Union Medical College, Beijing, 100730 China; 4 Clinical Immunology Center, Chinese Academy of Medical Sciences and Peking Union Medical College, Beijing, 100730 China

**Keywords:** Pancreatic cancer, Preclinical model, Precision medicine

## Abstract

Pancreatic ductal adenocarcinoma (PDAC) is an extremely lethal malignancy, with an average 5-year survival rate of 9% (Siegel RL, Miller KD, Jemal A. Ca Cancer J Clin. 2019;69(1):7-34). The steady increase in mortality rate indicates limited efficacy of the conventional regimen. The heterogeneity of PDAC calls for personalized treatment in clinical practice, which requires the construction of a preclinical system for generating patient-derived models. Currently, the lack of high-quality preclinical models results in ineffective translation of novel targeted therapeutics. This review summarizes applications of commonly used models, discusses major difficulties in PDAC model construction and provides recommendations for integrating workflows for precision medicine.

## Background

Pancreatic ductal adenocarcinoma (PDAC) is the fourth leading cause of cancer-related mortality in the United States. With the lowest 5-year relative survival rate among all cancer types and a contemporaneously increasing incidence rate [1], PDAC is predicted to become the second leading cancer killer by 2030 [2]. The poor prognosis of PDAC is attributed to the difficulty of early diagnosis, high rate of metastasis and resistance to chemotherapy. Molecular pathology studies identify *KRAS* activation in most PDAC patients, which is considered a key driver mutation of tumor progression. Other recurrent somatic mutations lead to the inactivation of *TP53*, *SMAD4*, and *CDKN2A* [3]. The frequency of these aberrations increases in higher grade pancreatic intraepithelial neoplasm (PanIN) lesions, indicating a stepwise accumulation of genetic alterations [4]. Multiomic profiling has enabled the classification of PDAC into subgroups with distinct tumor behavior, supporting the concept of patient stratification in the practice of precision medicine. Nonetheless, the low efficacy of targeted PDAC therapy suggests the significance of verification studies for patient-derived models.

**Fig. 1:**
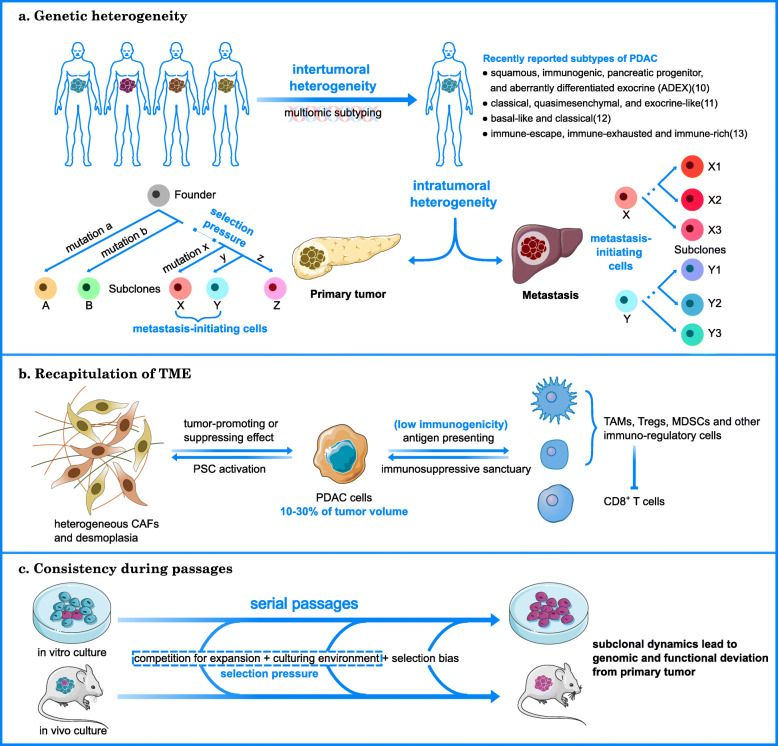
Difficulties in the preclinical modeling of PDAC. Figure was produced using Servier Medical Art (http://smart.servier.com/). **a** When establishing a preclinical model library, intertumoral heterogeneity at the multiomic level highlights the significance of cohort size, while intratumoral heterogeneity in temporal (subclonal evolution) and spatial (primary tumor and metastasis) dimension requires multiple sampling from individuals. **b** Complex tumor-stroma interactions and phenotypical heterogeneity of stromal components are major barriers to the recapitulation of the TME. Low immunogenicity and the immunosuppressive sanctuary of PDAC are also difficult for preclinical modeling. **c** Serial passaging of preclinical models enables the selection of malignant subclones of tumor cells, raising doubts on the application of cells from later passages. (10), (11), (12) and (13) denote reference [10–13], respectively. PDAC, pancreatic ductal adenocarcinoma; TME, tumor microenvironment; CAF, cancer-associated fibroblast; PSC, pancreatic stellate cell; TAM, tumor-associated macrophage; MDSC, myeloid-derived suppressor cell

Despite advancements in knowledge concerning the molecular mechanisms of PDAC tumorigenesis and progression, few preclinical discoveries have been successfully translated to clinical practice, suggesting insufficient recapitulation of critical tumor attributes in existing models [5]. Compared with other types of tumors, PDAC features a high level of inter- [3] and intratumoral [6, 7] heterogeneity that shapes the genomic landscape and affects therapeutic response. In addition, the tumor microenvironment (TME) of PDAC is characterized by extensive deposition of stromal components and strong immunosuppression [8]. These issues are usually underrepresented in model construction. Moreover, subclonal divergence from the primary tumor is introduced through serial passages [9], which is an indispensable process for in vitro culture and xenograft models (Fig. 1). Former studies have analyzed the strengths and weaknesses of individual models, while the appropriate integration of currently available models is required for the development of more reliable therapeutic strategies against PDAC. Therefore, this review summarizes the application of in vitro and in vivo preclinical models of PDAC and delineates their roles in each stage of precision medicine.

### Difficulties in the preclinical modeling of PDAC

#### Inter- and intratumoral heterogeneity

Major forms of intertumoral genetic heterogeneity include the genome-wide mutation landscape, transcriptomic characteristics, and epigenetic regulation patterns. It is estimated that > 90% of PDAC cases present with a *KRAS* mutation, while *TP53*, *SMAD4* and *CDKN2A* inactivation is presented by > 50% of PDAC cases [14]. Other recurrently mutated genes, including numerous druggable targets, are observed in only ~ 10% of cases [15]. These clinically relevant, infrequently mutated genes constitute a major aspect of intertumoral genetic heterogeneity, which leads to discordance between the results of clinical trials and basic research and encourages subtyping of PDAC through multiomics [10–13] (Fig. 1). Some of the identified subtypes have added instructive value to clinical practice. For example, whole-genome sequencing was used to classify PDAC into four groups (the stable subtype, locally rearranged subtype, scattered subtype and unstable subtype) according to the frequency and distribution of structural variations [14]. Genomic instability was discovered to be a putative biomarker of platinum-based chemotherapy and poly ADP-ribose polymerase (PARP) inhibitors. In addition, a recent multiomic analysis of 150 PDAC specimens conducted by the Cancer Genome Atlas (TCGA) Research Network [3] confirmed two tumor-specific subtypes: basal-like/squamous and classical/pancreatic progenitor. The latter was associated with better prognosis [12] and higher sensitivity to erlotinib [11] in earlier studies. Moreover, Wartenberg et al. [13] identified three subsets of PDAC with different levels of immune cell infiltration. Subsequent retrospective analysis of clinical data revealed longer overall survival (OS) and progression-free survival (PFS) for patients in the ‘immune-rich’ category than for patients in the ‘immune-exhausted’ category. However, these classification systems should still be evaluated before their extensive application. Most importantly, the sample sizes in these studies were insufficient to reflect the entire landscape of intertumoral heterogeneity [16].

It is widely accepted that any kind of treatment poses selective pressure on tumor cells, resulting in the dominance of resistant clones [7], which contributes to a worsened prognosis for patients. The de novo formation of these clones reflects the temporal dimension of intratumoral heterogeneity. Its spatial dimension is further classified into three subtypes: heterogeneity within a primary tumor, in metastasis initiating cells of the primary tumor, or within a metastatic lesion [6] (Fig. 1). To genetically resolve the evolutionary pattern of PDAC, systemic sequencing results were compared between metastatic and primary lesions [17]. The results showed that all the examined lesions shared identical mutations in driver genes. Moreover, high consistency among passenger mutations was found in metastatic cells. These findings indicated that major mutational events and chromosomal rearrangements in PDAC cells occurred early in primary lesions and followed a sequential trajectory leading to local invasion and metastasis. This step-by-step progression theory was recently challenged, as chromothripsis-induced copy number changes were shown to be profound transforming events during rapid changes in oncogenic and tumor suppressor gene expression [18]. Both linear and branched genetic phylogeny were identified in a genetically engineered mouse model (GEMM) [19]. Nonetheless, the unique distribution of theranostic mutations among primary and metastatic lesions does not weaken the significance of multiple samples used in preclinical model construction. For instance, multiple forms of *KRAS* mutations were discovered in the same PDAC sample [3], while epigenetic heterogeneity among intratumoral subclones was still under research. In addition, subgroups of malignant cells with distinct proliferative and migratory potentials were identified through single-cell RNA sequencing [20].

In translational research of targeted therapy, intertumoral heterogeneity sets the lower limit of sample size, while intratumoral heterogeneity undermines reliability of isolated sampling in individual patient [7]. Therefore, it is important to guarantee sufficient coverage of variant genotypes and intratumoral subclones of PDAC when building a preclinical model.

#### Recapitulation of the tumor microenvironment

PDAC tumor cells constitute 10–30% of the tumor volume. The remaining 80% are cancer-associated fibroblasts (CAFs), extracellular matrix (ECM) and immunosuppressive cells, which are closely related to the progression of cancer cells [21].

Fibroblasts are the principal regulator of desmoplasia formation [22]. In tumorigenesis, quiescent pancreatic stellate cells (PSCs) are activated by stimuli from preinvasive lesions. This initial wound-healing response evolves into comprehensive remodeling of the tumor microenvironment involving intricate tumor-stroma cross talk and the deposition of ECM components, ultimately establishing an immunosuppressive and chemoresistant sanctuary for PDAC cells (Fig. 1). However, fibroblasts pose great difficulty in establishing preclinical models, especially xenograft models, because of the involvement of host-derived fibroblasts. A study of a colorectal cancer (CRC) patient-derived xenograft (PDX) model [23] showed that the human stroma of tumor xenografts was entirely replaced by murine tissues during the second passage. GEMMs are the most widely used models to decipher and manipulate the regulatory mechanisms of TME [24] due to their endogenous stromal components. Apart from the discordant species origin of the tumor and stroma, the heterogeneity of the fibroblasts is another obstacle to TME modeling (Fig. 1). Noticeably, the expression of activation markers classifies CAFs into subtypes with distinct secretome and cellular behavior [25]. For instance, the tumor-promoting alpha smooth muscle actin (αSMA)-negative/fibroblast activation protein (FAP)-positive CAF population expresses higher levels of chemoresistance-mediating interleukin-6 (IL-6), [26] and immunosuppression-mediating CXC chemokine receptor 2 (CXCR2) ligands [27] than the tumor-suppressing αSMA^+^/FAP^−^ CAF population. Mechanistically, the αSMA^+^ population contributes to high interstitial pressure [22], which inhibits drug transportation and immune cell infiltration. In addition to the refined characterization and subtyping of tumor cells, more efforts need to be focused on the heterogeneity of fibroblasts. Retrospective analyses of clinical data [28, 29] revealed that an extensive stroma was correlated with poor survival, but stroma depletion therapy led to divergent outcomes, either improving [30] or worsening [31, 32] the prognosis. The fibroblast profiling of individual patients may provide crucial clues for accurate stroma manipulation through immunologic strategies, including the use of chimeric antigen receptor T (CAR T) cells [33].

Immune compartments are among the most promising TME targets in anticancer research. The resistance of PDAC to mono-immunotherapy has led to the construction of a preclinical model for combination therapy screening [34]. Nonetheless, each in vivo PDAC model has specific limitations in the recapitulation of low PDAC immunogenicity and the immunosuppressive TME. Among xenograft models, syngeneic cell line transplantation into immunocompetent mice and the construction of ‘humanized mice’ [35] maintain the functional immune system of the host. These models can be exploited to study how exogenous PDAC adapts to host immune pressure [36] but fail to represent the induction of immune privilege during tumor development. GEMMs are acknowledged as better for predicting immunotherapy response due to their autochthonous tumors and extensively integrated immunosuppressive mechanisms. For instance, the depletion of Tregs in orthotopic xenograft models enhances the antitumor effect of CD8^+^ T cells [37] but has a minor influence on CD8^+^ T cell recruitment in GEMMs [38]. However, the low mutation burden of GEMMs leads to deficiency of neoantigen expression [39], which is the main T cell target in long-term survivors of PDAC [40].

In translational medicine, cases of failure in stroma-targeted therapy are exploited to decipher resistance mechanisms and identify biological markers. This process calls for the accumulation of preclinical evidence and advances in PDAC modeling technology.

#### Consistency during passaging

For all kinds of preclinical models, passaging is an inevitable process that confers selective pressure on the tumor culture, leading to functional deviation from the primary tumor. For instance, later passages of the PDX cells tend to be more proliferative, aggressive, and easier to metastasize [41]. These changes are deeply rooted in the subtle drift of the genetic landscape. Dynamic changes causing various genomic instabilities are documented during PDX passaging [9], including copy number alteration (CNA), which is associated with tumor progression [42]. Monitoring 1110 PDX models across 24 tumor types [43] revealed model-acquired an average of 12.3% CNA in the genome within four passages. Clonal selection, but not genomic instability, was suggested to be the source of this CNA dynamic. Coleman et al. [44] compared the proteomic patterns of primary and passage 1 PDX of PDAC cells based on liquid chromatography-mass spectrometry (LC-MS)/mass spectrometry (MS) data, and the results showed that 143 human-specific proteins were differentially expressed. It was remarkable that most of these alterations were enriched in pathways related to tumor proliferation, invasion, angiogenesis and stemness [45]. The extent of genetic drift greatly depends on the selection power of the culture environment. For example, patient-derived cell lines (PDCLs) of PDAC preserve oncogenic mutations and their overall transcriptional profile through as many as 40 passages when cultured with collagen matrix in vitro [46].

Genetic drift leads to subclonal dynamics. Subclones consist of cells with similar proliferation rates. Nguyen et al. [47] used DNA barcoding to reveal different growth patterns of each subclone during passaging, which corresponded to a gain or loss of dominance. Noticeably, there was no established correlation between genetic and functional subclones, as high-coverage whole-genome sequencing of CRC xenografts [48] indicated that tumor clone-initiating cells were genetically heterogeneous. From an evolutionary perspective, systemic selection generates dominant subclones during passaging, with the mutation profile of the entire tumor gradually converging at specific subclones [49]. Selection pressure is established not only because of intratumoral competition for maximum expansion speed but also because of adaptation to the culture environment. In addition, selection bias randomly resets the subclone constitution between successive passages [9] (Fig. 1). Poor genetic and functional consistency of PDAC models stress the significance of using early passages in preclinical studies.

### Characterization and application of common PDAC models

#### Cell lines

Cell lines are homogeneous and easy to propagate and therefore suitable for high-throughput bioinformatics studies (Table 1). For example, proteomic analysis and subsequent immunoprecipitation–mass spectrometry (IP–MS) assay of the MIA PaCa2 cell line [50] revealed that the leukaemia inhibitory factor (LIF) receptor was a therapeutic target of tumor-stroma interactions. In addition, cell lines also provide great convenience for genetic manipulation (Table 1). For instance, McDonald et al. [51] discovered the key regulatory role of carbonic anhydrase 9 (CA9) in the adaptation of PDAC cells to hypoxic environments by knocking down or chemically inhibiting CA9 in GEMM-derived cell lines. Besides, the transplantation of cell lines into immunodeficient mice enables in vivo verification of target-specific interventions. Although subcutaneous transplantation is easier to scale and reproduce, the orthotopic model shares more genetic and metabolic similarities with naturally growing tumor [52]. Moreover, direct or indirect in vitro co-culture of PDAC cells with representative stromal cells offers a glimpse of complex tumor-stroma cross-talk (Table 1). For example, culture of PSCs with PDAC cells [53] suggested PDAC-derived galectin-3 promote PSC secretion of proinflammatory factors. Similar methodology was applied to identify a panel of TME regulators [54–56]. However, 2D co-culture is incomplete in modeling tumor heterogeneity, structure, intercellular contact, or gene expression. Function of identified mediators still needs validation in PDO [57] or PDX [58] models.

**Table 1:** Characteristics of common preclinical models of PDAC

	Cell lines	Organoids	PDX	GEMM
Intertumoral heterogeneity	Between tumor cell lines (database available)	Between sampled cases	Between sampled cases	–
Intratumoral heterogeneity	–	Depend on sampling region	Depend on sampling region	+++
Tumor-stroma interaction	+ (Co-culture)	++ (Co-culture)	++	+++
Consistency during passages	+	++	+++	+++
Expansion	+++	+++	++	+
Genetic manipulation	+++	+++	+ (Before transplantation)	++
High throughput screening	+++	+ (Costly)	+ (Costly)	–
Success rate of initiation	++	++	++	+++
Cost	$	$$	$$	$$$
Time	1 month or less	1–2 months	More than 6 months	More than 6 months
Other strengths	Standardized across laboratories	Cultured from diverse cells or tissue	Mirror patient response to targeted therapy	Model all stages of tumor progression
Other weaknesses	Finite number of widely-available cell lines	Lack high quality clinical trials	Lack infiltrating immune cells, Loss of original stroma, Only represent resectable lesion	Mouse genomic background different from human

*PDX* patient-derived xenograft, *GEMM* genetically engineered mouse model

+++ denotes good; ++, medium; +, limited; −, not suitable

Poor consistency between passages undermines the predictive power of the cell line model in clinical practice (Table 1). For example, the deviation of genome-wide CNAs [43] and variations in the DNA methylation pattern [59] were both found in newly established PDAC cell lines, indicating in vitro culture as a possible driving force in genetic and epigenetic aberrations. However, collectively assembled cell lines are qualified to model basic tumor behavior. According to parallel sequencing results of 41 PDAC cell lines in Cancer Cell Line Encyclopedia (CCLE) [60], chromosomal copy number, gene expression patterns and point mutation frequencies of cell lines were strongly correlated with primary PDAC, as indicated by Tumorscape, Expression Project for Oncology (expO) and Catalogue of Somatic Mutations in Cancer (COSMIC) data sets, respectively. In addition, transcriptional profile-defined classical and quasimesenchymal subtypes were identified in cultured cell lines [11], with subtype-specific *KRAS* dependence and drug response maintained. As an important part of the intratumoral heterogeneity of PDAC, cancer stem cells (CSCs) were also identified in human PDAC cell lines through real-time imaging [61].

#### Organoids

Construction of organoids requires 3D culture of resected tumor specimens, biopsy samples or even pluripotent stem cells [62]. Organoids can be propagated, passaged and cryopreserved. The integrated culture medium formula for murine and human PDAC was suggested by Clever and Tuveson laboratories [63], who orthotopically transplanted PDAC organoids to immunocompromised mice to generate PanIN-like preinvasive lesions, creating a promising model of tumor progression.

Organoids and cell lines have shared advantages and applications. They both allow high-throughput drug screening (HTDS) (Table 1), especially after large-scale production of homogenous organoids, which can be carried out economically [64, 65]. Compared to cell lines, organoids show greater similarity with primary tumors (Table 1). For example, systemic sequencing discovered a strong correlation between organoids and tumors of origin in individual-specific mutations [66]. Concordant expression shifts in cancer-related pathways were also documented. To test the clinical relevance of organoids, a patient-derived organoid (PDO) library [67] was established with tumor samples derived from 66 PDAC patients receiving chemotherapy. Patient prognoses revealed high consistency with the drug response of the corresponding organoids. Longitudinal sampling from a single patient successfully predicted his acquisition of chemoresistance during disease progression. Even subtle intratumoral heterogeneity was faithfully recapitulated, as single cell-derived organoids of the same CRC patient showed distinct drug sensitivity due to scattered sampling [68]. In a direct comparison of drug response between patient-derived ovarian cancer cell lines and the corresponding organoids [69], fewer organoid cells died after 72 h of drug administration but showed higher levels of cell death after drug removal, suggesting better recapitulation of chemoresistance and drug-scavenging effects. These studies indicate that the genotype-phenotype relationship is better preserved in 3D culture [70]. Organoids bridge the gap between cell lines and a PDX model in terms of the identification of main targets from a complex mutational landscape and aberrant epigenetic regulation [71, 72]. The application of organoids is further suggested due to the construction of PDO biobanks that can integrate preclinical modeling with genetic information and clinical background [62]. Besides, co-cultured organoids promote study of tumor-stroma interactions (Table 1). Öhlund et al. [25] reconstructed desmoplastic stroma and reestablished heterogeneous CAFs in co-cultures of murine PSCs and PDAC organoids. In addition, co-culture of peripheral blood lymphocytes [73] enriched the infiltration of reactive T cells in the TME of CRC and non-small cell lung cancer (NSCLC) organoids. This platform enabled the prediction of an immunotherapy response and produced highly selective T cells for adoptive T cell transfer.

#### Patient-derived xenografts

Fu et al. [74] devised the first PDX model of pancreatic cancer by transplanting histologically intact pancreatic cancer specimens from five patients into athymic nude mice. Kim et al. [75] improved the protocol by changing the host to immunodeficient nonobese diabetic/severe combined immunodeficiency (NOD/SCID) mice, which increased tumor-forming efficiency.

Due to the high time and resource costs in their construction [75] (Table 1), PDX models are mostly used for guiding personal treatment. The concept of a co-clinical model trial [76] underlines parallel treatment of patient and xenograft mice, which is supported by the solid consistency of clinical outcomes (Table 1). Apart from precision medicine, the PDX platform can be adopted in novel translational anticancer research, as it allows simulation of versatile tumor phenotypes, including local invasion, metastasis and drug resistance. For instance, a switchable CAR T-cell system [77] was shown to induce remission in a PDX model of a stage IV PDAC patient with equal efficacy and lowered off-target rate compared to conventional CAR T cell therapy. The TME of the PDX model features tumor and stromal cells of different species of origin. Respective multiomic sequencing elucidated the mechanism of mutual adaptation [78]. In addition, HTDS on a large-scale PDX cohort was used to identify prognosis-associated biomarkers and facilitate the efficacy of targeted therapy [79].

There are still many limitations of PDX models (Table 1). First, the sampling process of patient tumor specimens, especially fine-needle biopsies, leads to poor representations of intratumoral heterogeneity. Second, PDX models lack infiltrating immune cells in the TME. The humanized mouse model [80] partially reconstructed the host immune system by introducing patient-derived CD34^+^ hematopoietic stem cells (HSCs). Currently, more efforts are devoted to developing novel GEMM-expressing cytokines for HSC activation [81]. Third, stroma replacement with host components results in genetic and functional drift from the primary tumor [82]. Later passages were found to be more sensitive to pharmacological treatment [9]. Fourth, PDX models are difficult to genetically manipulate. Molecular interventions need to be conducted in patient-derived tumor cells before transplantation.

#### Genetically engineered mouse models

The first GEMMs, known as KC mice [83], presented with the conditional expression of *Kras*^*G12D*^ in epithelial cells of pancreatic lineage. They mimicked PDAC tumor progression from PanIN to local invasive lesions. To promote malignancy of the pretumor lesions, concomitant *Kras*^*G12D*^ and *Trp53*^*R172H*^ mutations were introduced in KPC mice [84], which reconstructed the whole spectrum of tumor progression. For example, KPC mice < 10 weeks, 10–12 weeks, > 12 weeks old were enrolled in chemoprevention (preventing PanIN formation), early (preventing PanIN progression) and late interventional (circumventing metastasis) studies, respectively [85]. In addition, KPC mice recapitulated the autologous TME and showed great potential in stroma-targeted therapy development (Table 1). For instance, Pegvorhyaluronidase alfa (PEGPH20) [86], an enzymatic agent of hyaluronic acid (HA), restored interstitial fluid pressure and re-expanded the microvasculature in the PDAC TME. The combination of PEGPH20 and gemcitabine significantly promoted tumor regression and overall survival of the KPC mice. A phase II clinical trial [87] corroborated the efficacy of PEGPH20 in promoting the PFS of metastatic PDAC patients. Currently, most GEMMs were inoculated with targeted mutations through bacteriophage-derived Cre recombinase prior to or simultaneous to PDAC initiation, resulting in a disturbance to the natural tumorigenesis process. A recently devised dual recombinase system (DRS) [88] allowed the sequential expression of *Kras*^*G12D*^ and target mutations in pancreatic cells. This model could be exploited to dissect the genetic events of tumorigenesis in a stage-specific manner and to validate therapeutic targets in both PanIN and invasive PDAC. For example, the chromatin remodeler Brahma related gene 1 (Brg1) was critical for PanIN and PDAC formation in the DRS [89], as shown by induced *Brg1* deletion leading to widespread apoptosis of PanIN cells. Introducing more driver mutations to a DRS may broaden its application to therapies targeting malignant PDAC.

Despite their broad applications, GEMMs have several limitations. The selection of driver mutations leads to the loss of PDAC intertumoral heterogeneity. Deviation of the human and mouse genomes also requires careful interpretation of preclinical study results. The generation of GEMMs is time- and resource-consuming, making it unsuitable for high-throughput sequencing or drug screening (Table 1). Moreover, the use of GEMMs does not guarantee that the primary tumor burden or extent of the metastasis is consistent among the enrolled mice [85], as multiple factors may lead to bias in therapeutic outcomes and survival.

### Choice of PDAC models in preclinical studies and precision medicine

#### Target screening in basic cancer research

**Fig. 2:**
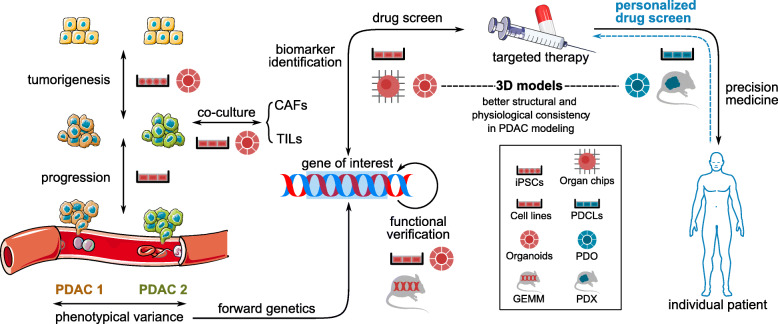
Choices of preclinical models for use in preclinical studies and precision medicine of PDAC. Figure was produced using Servier Medical Art (http://smart.servier.com/). Clonal and expandable models are used in basic researches to decipher the molecular mechanism of distinct tumor behavior. Cell line or organoid-based co-culture of PDAC with TME components could be exploited in study of tumor-stoma interaction. As a core target of translational research, the prediction of genotype-drug response mainly relies on model-specific high-throughput sequencing and drug screening. Recently, more focus has been directed to personalized therapy development based on patient-derived cell lines, organoids and xenografts. 3D models excel 2D cell culture in structural and physiological consistency with naturally growing tumor and will play a more important role in precision medicine of PDAC. Of all the preclinical models, use of early passages could minimize genetic and functional drift away from the primary tumor. PDAC, pancreatic ductal adenocarcinoma; CAF, cancer-associated fibroblast; TIL, tumor infiltrating lymphocyte; iPSC, induced pluripotent stem cell; GEMM, genetically engineered mouse model; PDCL, patient-derived cell line; PDO, patient-derived organoid; PDX, patient-derived xenograft

Basic cancer research focuses on genotypical and phenotypical manifestations of PDAC cells. The concept of forward genetics predominates this stage with respect to studying the molecular mechanism of distinct clinical phenomena. High-throughput sequencing is generally needed to dissect the aberrant pathways and ultimately identify target molecules of interest. Therefore, these studies require the use of clonal and possibly expandable models. Specific choices are made according to classification of tumor stage. Namely, organoids and patient-derived induced pluripotent stem cells (iPSCs) are widely used in modeling tumorigenesis, while established cell lines are more commonly exploited to recapitulate tumor progression (Fig. 2).

PDO allows modeling of various stages of PDAC [62], either through culturing tumor samples of different malignancy stages or the introduction of driving mutations into normal tissue-derived organoids [90]. Comparison between organoids of different malignant stages elucidates key regulators in tumor progression. For example, Roe et al. [91] compared organoids of primary tumors and metastatic lesions from the same KPC mice and discovered large-scale activation of enhancers associated with foregut endoderm development in the metastatic organoids. Besides, Seino et al. [90] analyzed transcriptomic data of 39 PDAC PDOs and found a correlation between higher Wnt independency and increasing metaplasia. Apart from organoids, iPSCs could also generate diverse histological components from PanIN-like lesions to invasive PDAC through injection into immunodeficient mice. Compared with organoids, the iPSC-based model is more powerful for dissecting the role of oncogenic mutations in cell lineage determination and tumorigenesis. iPSCs can also be 3D cultured to model histological layers and progenitor cell migration at various stages of tumorigenesis. For example, secreted proteins in conditioned medium from iPSC-derived PDAC organoid cultures provided clues of candidate biomarkers for the early detection of PDAC [91]. The greatest advantage of organoids and iPSCs is their possible use for large-scale expansion, deep sequencing and genetic manipulation.

Despite the inability to model early tumor development, established cell lines provide a practical and affordable platform to dissect the molecular mechanism behind phenotypical variances concerning growth rate, metastatic tendency, stroma formation upon transplantation and drug resistance. For instance, a comparison of the expression profile between chemosensitive and chemoresistant PDAC cell lines [92] revealed differential expression of epithelial-to-mesenchymal transition (EMT)-related genes, a finding that was confirmed by real-time PCR. Similar methods were exploited to elucidate key mRNAs involved in cancer stem cell regulation [93]. Currently, more target searching strategies are being developed to excavate these genetic resources. For example, a genome-wide screen for proliferation-dependent genes in 216 cancer cell lines [94] was carried out using a lentivirally delivered shRNA library. Moreover, a genomic data-based algorithm [95] was proposed for use in discovering synthetic lethality of specific genes in cancer cell lines.

Identification of target molecule is one of the most challenging steps in translational cancer research. A large collection of homogeneous and expandable models is favored in this process so as to overcome heterogeneity of pancreatic cancer. Compared to cell lines, PDOs and iPSCs are more resource-demanding, but excel in consistency with primary tumor. Most importantly, they are all suitable for genetic manipulation and in vitro verification of potential target, while in vivo phenotypical assay could be based on xenograft model or GEMM.

#### Biomarker identification in pharmacogenomic studies

In order to explore the translational value of a druggable target, HTDS is usually carried out which mimics physiological drug delivery and allows repeatable analysis of key tumor phenotypes. Cell lines, organoids and organ chips are most commonly exploited in HTDS (Fig. 2). The concept of pharmacogenomic database [96] links multiomic sequencing and drug response data of preclinical platform. Integrated analysis enables computational prediction of treatment efficacy based on the genetic and epigenetic signatures of individual patients.

Cell line panels have been acknowledged as a mature platform to assess the clinical utility of investigational anti-cancer drugs and to discover predictive biomarkers. Databases such as CCLE [60] and Genomics of Drug Sensitivity in Cancer (GDSC) [97] use endpoints including genomic, transcriptomic, metabolomic and proteomic aberrations to build pharmacogenomic algorithms. Short-term cell line culture could supplement the database to enhance the depth of the sequencing and increase the genomic consistency with naturally growing tumors [98]. A comprehensive framework [99] is developed to guarantee the credibility of the cell lines and rule out misidentified genes or cross-contamination based on single nucleotide polymorphism (SNP) genotyping, short tandem repeats (STR) profiling and cross-species PCR. Moreover, pooled screening of cancer cell line mixtures [100] was developed to analyze the growth rate of each labeled cell line in xenografts, thereby enabling in vivo drug sensitivity studies. However, lack of 3D tissue structure in cell line cultures may result in biased predictions of drug response. Recently PDAC organoid-based HTDS [65] was conducted through combination of cell-repellent surface and bioprinting technology. Another novel 3D culture system [101] yielded cancer tissue-originated spheroids from incompletely dissociated tumor fragments. The high recovery and purification rate of tumor cells as well as better maintenance of intercellular interaction made these spheroids compatible with HTDS. Future development of HTDS platform focuses on multicellular architecture, tissue interfaces and mechanical forces involved in tumor growth. In microfluidic organs-on-chips [102], cells could survive for months with nutrient sources delivered through endothelium-lined vascular structures. This system can be exploited to model cell migration, mimic the concentration gradient of chemicals, dissect stroma-related pharmacological mechanisms and recapitulate novel pharmacokinetic processes. Moreover, construction of body-on-chip model through combination of organ chips may facilitate the study of metastasis-related tumor behavior.

Multiomic profiling and HTDS are two main steps of pharmacogenomic study, with the final aim of predicting efficacy of targeted therapy in individual patient. HTDS requires not only genetic or phenotypical, but structural and even physiological consistency between preclinical models and naturally growing tumors. Cell line-based platform has built systemic algorithm in biomarker identification, which provides a solid foundation for the prosperity of 3D modeling in pharmacogenomic research.

#### Personalized drug screen

Genotype-drug response prediction is the mainstream of precision medicine practices. However, it is still likely that mutation profiles of individual tumor provide incorrect guidance for treatment. For instance, basic research of PDAC linked deficient levels of the chromatin remodeling BRG1-associated factors (BAF) with sensitivity to an enhancer of zeste homolog 2 (EZH2) inhibitor [103]. However, the cell lines derived from a wild-type patient manifested higher sensitivity to EZH2 inhibition than cell lines harboring mutations in chromatin remodelers [104]. Genetic drift in preclinical models is the most possible reason, which underscores the significance of personalized drug screen on patient-derived preclinical models (Fig. 2).

PDCL-based drug screening leads to the discovery of exceptional responses to certain agents that are negligible in established cell lines and thus not strongly predicted. For example, few PDAC patients demonstrate sensitivity to mitogen-activated protein kinase kinase (MEK) inhibitors administered as single-agent therapy [105], but one PDCL [104] demonstrated significant suppression of the cell cycle and induced apoptosis upon MEK inhibitor administration. This sensitivity was confirmed in the PDX model, which showed suppressed tumor growth. Another exclusive strength of PDCL-based drug screening lies in combination therapy designs. This screening could be carried out on a microfluidics platform [34] based on patient biopsy sample-derived live tumor cells without the need for intermediate culturing, which shortened the time span of model construction and enabled the control of possible genetic drift. The drug response predicted on this platform was very consistent with the results from the xenograft studies and clinical outcomes. Despite relatively high cost, PDX and ex vivo models provide more reliable results in individual drug screening. The rationality of the ‘one animal per model per treatment’ approach was proven by a retrospective analysis of 2138 PDX mice receiving 440 treatment plans [79]. For each therapy, 95% of the individual response results were consistent with the collective outcomes. In addition, a similar distribution of results was found in this ‘PDX encyclopedia’ and patients in independent phase II clinical trials. Consistency between PDX models and patients at both the population and individual levels indicate a promising application of PDX models to targeted drug development and precision medicine, respectively. Although large-scale PDX screening is sometimes impractical for researchers, ex vivo culture systems can serve as qualified surrogates. A personalized tumor ecosystem [106] was engineered by culturing explant tumor slices together with matched tumor matrix proteins and autologous serum from the patient. This system captured more details of intratumoral heterogeneity, including clonal diversity of the tumor cells and tumor-stroma spatial distribution, than other in vitro preclinical models.

In comparison to conventional patient subgrouping strategies, verification of the treatment efficacy in personalized preclinical model lowers the possibility of genetic drift and resultant pitfalls in targeted therapy. This process will carry more weight in the evolving rationale for the use of precision medicine.

## Conclusion

The last decade witnessed tremendous advancements in the knowledge of PDAC tumor progression and novel tumor-stroma interactions. Nonetheless, there has been no significant improvement in the prognosis for PDAC patients. Countless failures of targeted therapy in phase I/II clinical trials reflect the problems with preclinical models. Poor recapitulation of drug responses and therapy resistance calls for precision modeling both in systemic platform confluence and individual model construction. In summary, the heterogeneity of PDAC requires multiple temporally and spatially obtained samples from a large cohort in any translational research. Use of early cell passages for preclinical models could minimize genetic and functional drift away from the primary tumor. In an integrated workflow of precision medicine, target molecules are first identified through bioinformatics studies based on cell lines, iPSCs or organoids. Functional verification is then conducted through genetic engineering or chemical manipulation. The roles of candidate genes as potential biomarkers are established after cell line, organoid or organ chip-based high-throughput drug screening and careful evaluation of clinical relevance. Efficacy verification of the patient-derived model is still required after genomic subtyping. The appropriate choice of an existing model in these stages narrows the systemic error of patient outcome prediction (Fig. 2).

However, the errors of PDAC modeling in precision medicine can only be absolutely circumvented by the creation of a primarily humanized platform. Through interdisciplinary collaboration, iPSCs and regenerative biomaterial technology have shown great potential in tissue-engineered mouse models [107], where spatial and functional interactions of patient-derived tumor cells and tissue compartments are closely recapitulated. The predictive potential of these innovative models should be tested with computational integration of clinical data at different points of model development [107]. If there is inconsistency regarding genetic profiles, histopathology, tumor progression profile or therapy response, the model needs to be redesigned and re-evaluated. The evolution of preclinical models will contribute to rapid translation of preclinical results.

## Data Availability

Not applicable.

## Abbreviations

αSMA: Alpha-smooth muscle actin
BAF: BRG1-associated factors
CA9: Carbonic anhydrase 9
CAF: Cancer-associated fibroblast
CAR T: Chimeric antigen receptor T cell
CCLE: Cancer Cell Line Encyclopedia
CNA: Copy number alteration
COSMIC: Catalogue of Somatic Mutations in Cancer
CRC: Colorectal cancer
CSC: Cancer stem cell
CXCR2: CXC chemokine receptor 2
DRS: Dual recombinase system
ECM: Extracellular matrix
EMT: Epithelial-to-mesenchymal transition
expO: Expression Project for Oncology
EZH2: Enhancer of zeste homolog 2
FAP: Fibroblast activation protein
GDSC: Genomics of drug sensitivity in cancer
GEMM: Genetically engineered mouse model
HA: Hyaluronic acid
HSC: Hematopoietic stem cell
HTDS: High-throughput drug screening
IL-6: Interleukin-6
IP-MS: Immunoprecipitation-mass spectrometry
iPSC: Induced pluripotent stem cell
LC-MS: Liquid chromatography-mass spectrometry
LIF: Leukaemia inhibitory factor
MDSC: Myeloid-derived suppressor cell
MS: Mass spectrometry
NOD/SCID: Nonobese diabetic/severe combined immunodeficiency
NSCLC: Non-small cell lung cancer
OS: Overall survival
PanIN: Pancreatic intraepithelial neoplasm
PARP: Poly ADP-ribose polymerase
PDAC: Pancreatic ductal adenocarcinoma
PDCL: Patient-derived cell line
PDO: Patient-derived organoid
PDX: Patient-derived xenograft
PEGPH20: Pegvorhyaluronidase alfa
PFS: Progression-free survival
PSC: Pancreatic stellate cell
SNP: Single nucleotide polymorphism
STR: Short tandem repeats
TAM: Tumor-associated macrophage
TCGA: The Cancer Genome Atlas
TIL: Tumor infiltrating lymphocyte
TME: Tumor microenvironment
